# Comparison of the clinical and pathological characteristics of ultrasound-guided biopsy for breast masses and non-mass lesions between 16-gauge spring-loaded core needle biopsy and 12-gauge spring-loaded vacuum-assisted biopsy

**DOI:** 10.1007/s10396-022-01279-3

**Published:** 2023-01-16

**Authors:** Yuka Yashima, Tomoyuki Fujioka, Kazunori Kubota, Mio Mori, Arisa Sato, Goshi Oda, Tsuyoshi Nakagawa, Iichiroh Onishi, Mayuko Tanaka, Ukihide Tateishi

**Affiliations:** 1 Department of Diagnostic Radiology, Tokyo Medical and Dental University, 1-5-45 Yushima, Bunkyo-Ku, Tokyo, 113-8510 Japan; 2 Department of Radiology, Dokkyo Medical University Saitama Medical Center, 2-1-50 Minamikoshigaya, Koshigaya, Saitama 343-8555 Japan; 3 Department of Surgery, Breast Surgery, Tokyo Medical and Dental University, 1-5-45 Yushima, Bunkyo-Ku, Tokyo, 113-8510 Japan; 4 Department of Diagnostic Pathology, Tokyo Medical and Dental University, Tokyo, 113-8510 Japan; 5 Department of Radiology, Ome Municipal General Hospital, Tokyo, 198-0042 Japan

**Keywords:** Ultrasound, Core needle biopsy (CNB), Vacuum-assisted biopsy (VAB), Breast cancer, Positive predictive value 3 (PPV3)

## Abstract

**Purpose:**

To retrospectively compare the clinical and pathological characteristics of breast masses and non-mass lesions that underwent ultrasound (US)-guided 16-gauge spring-loaded core needle biopsy (CNB) or 12-gauge spring-loaded vacuum-assisted biopsy (VAB).

**Methods:**

We retrospectively compared the results from US-guided diagnostic breast biopsy performed with a 16-gauge CNB (Magnum™) or a 12-gauge VAB (Celero^®^). The patients’ backgrounds and pathological features for each device were examined.

**Results:**

In 453 patients with 500 lesions, 373 lesions underwent CNB and 127 underwent VAB. The positive biopsy rate (positive predictive value 3) was significantly higher for VAB (92/127; 72.4%) than for CNB (231/373; 61.9%) (*P* = 0.032). Non-mass lesions were biopsied more frequently with VAB (57/127; 47.4%) than with CNB (27/378; 7.14%) (*P* = 0.000). The upgrade rate from high-risk to malignant lesions was significantly higher for CNB (5/19; 26.3%) than for VAB (1/8; 12.5%) (*P* = 0.043). There were five (1.34%) specimen failures with CNB and one (0.78%) with VAB, 18 (4.82%) re-biopsies with CNB and three (2.36%) with VAB, and 11/21 (52.4%) upgrades from ductal carcinoma in situ (DCIS) to invasive ductal carcinoma (IDC) with CNB and 11/30 (36.7%) with VAB. Although these rates tended to be higher with CNB than with VAB, the difference was not significant.

**Conclusion:**

Although VAB had a significantly higher rate of non-mass lesion biopsies, the upgrade rate from high-risk to malignant lesions was significantly lower for VAB than for CNB. US-guided 12-gauge spring-loaded VAB may be more appropriate for biopsy of non-mass lesions.

## Introduction

Ultrasound (US) is a major noninvasive diagnostic tool for evaluating breast lesions and is widely used in screening, differential diagnosis, preoperative staging, treatment-response assessment, and post-treatment surveillance [1, 2]. Besides breast cancer, malignant tumors of the breast include metastatic tumors, malignant lymphomas, and sarcomas. On the other hand, benign tumors include fibroadenomas, intraductal papillomas, and mastopathy [3]. Breast lesions are categorized according to the Breast Imaging Reporting and Data System (BI-RADS) on the basis of the overall findings of shape, border, size, and internal and posterior echography [4]. Benign and malignant lesions typically can be differentiated using US alone, but the imaging findings of the two types often overlap and need to be examined histologically. Reportedly, most breast cancers are visualized as masses on US, but ductal carcinoma in situ (DCIS) and lobular carcinoma are often detected as non-mass lesions [5, 6]. A non-mass lesion is defined as a hypoechoic area without an associated mass and is an important finding. However, in BI-RADS, non-mass lesions are clearly defined on magnetic resonance imaging but not on US [4].

In recent years, there have been remarkable advances in chemotherapy and molecular targeted therapy for breast cancer, allowing a variety of treatment options for each cancer subtype [7]. To provide patients with appropriate treatment, breast lesions must not only be diagnosed as benign or malignant but also pathologically assessed for malignancy and biomarkers. US-guided sampling is widely used for tissue diagnosis of breast lesions, because it is minimally invasive and safe. There are two main types of image-guided tissue sampling: core needle biopsy (CNB) and vacuum-assisted biopsy (VAB). VAB is more invasive than CNB, although it is generally possible to obtain a larger amount of tissue [8]. There are also a number of devices available from different vendors. Sample volumes vary widely between these methods and devices [9].

VABs can be divided into two categories: console and handheld. Handheld VABs are further divided into those that require a power supply and those that do not; the latter is the Celero^®^ spring-loaded system (Hologic Inc., Bedford, MA), which is a relatively new device that has been in use in Japan since October 2014 [9].

Several studies have compared the usefulness of US-guided CNB and VAB in histological diagnosis of breast lesions [10, 11], but no study has compared US-guided spring-loaded CNB with spring-loaded VAB. It has not been fully discussed whether spring-loaded VAB exhibits the same diagnostic performance as VABs that require a power supply.

The study aim was to retrospectively compare the clinical and pathological characteristics of patients with breast lesions, including both mass and non-mass lesions subjected to US-guided spring-loaded 16-gauge CNB or 12-gauge spring-loaded VAB. Furthermore, the patient backgrounds and pathological characteristics associated with each device were examined, and the role of US-guided CNB and VAB in the diagnosis and management of patients with breast lesions was discussed.

## Materials and methods

The study protocol was conducted in accordance with the ethical guidelines of the Declaration of Helsinki and approved by the Ethics Committee of our institution (approved 08 May 2021, approval ID: M2020-339). Our Ethics Committee waived the requirement for written informed consent.

### Study population

The inclusion criteria were: (a) patients who underwent US examination and were diagnosed with a breast lesion, and (b) patients who underwent US-guided spring-loaded 16-gauge CNB or 12-gauge spring-loaded VAB of the breast lesion between February 2016 and November 2019. Patients undergoing chemotherapy or hormone therapy were excluded. Patients who had undergone US examinations at other facilities were excluded to standardize the image quality.

After reviewing the radiology reports database and clinical records at our institution, two radiologists (with 4 and 11 years of imaging experience) retrieved the US images and findings, clinical information, and histopathological results of patients who underwent US-guided 16-gauge CNB or 12-gauge VAB during the study period. Breast lesions were classified into two categories: mass and non-mass lesions. A mass lesion was defined as a space-occupying lesion depicted in two different projections. A non-mass lesion was defined as an identifiable area of echotexture altered relative to that of the surrounding breast tissue but that did not conform to a mass shape [12]. A comprehensive final diagnosis of benign or malignant condition was made on the basis of imaging findings, histopathological results, and subsequent disease course. Patients with benign or high-risk lesions diagnosed based on US-guided sampling who were not followed up for > 1 year were excluded.

Immediate post-procedural complications were assessed by the radiologist who performed the biopsy. Delayed complications were evaluated by the breast surgeon 2 weeks after the biopsy when the patient visited to discuss biopsy results.

### US examinations and US-guided biopsy

Each examination was performed by one of five board-certified radiologists with 5–20 years of experience in breast US using either an Aplio XG or an Aplio 500 scanner, both with a PLT-805AT 8.0-MHz linear probe (Toshiba Medical Systems, Tochigi, Japan).

Patients were placed in the supine position on the examination bed with their arms down. Transverse and longitudinal static images were obtained, and the maximum breast lesion diameter was measured in B-mode. Subsequently, the radiologist evaluated the breast lesion and decided whether or not to perform US-guided sampling. Generally, BI-RADS 4 or 5 lesions were biopsied, but BI-RADS 3 lesions were sometimes biopsied at the request of the patient or surgeon. The choice between US-guided CNB and VAB was at the discretion of the radiologist on the basis of the BI-RADS category and the characteristics of the lesion. Basically, CNB was selected because of its low invasiveness and low cost, while VAB was selected when it was expected to be difficult to obtain a specimen or an abundant volume might be required for pathological diagnosis.

**Fig. 1:**
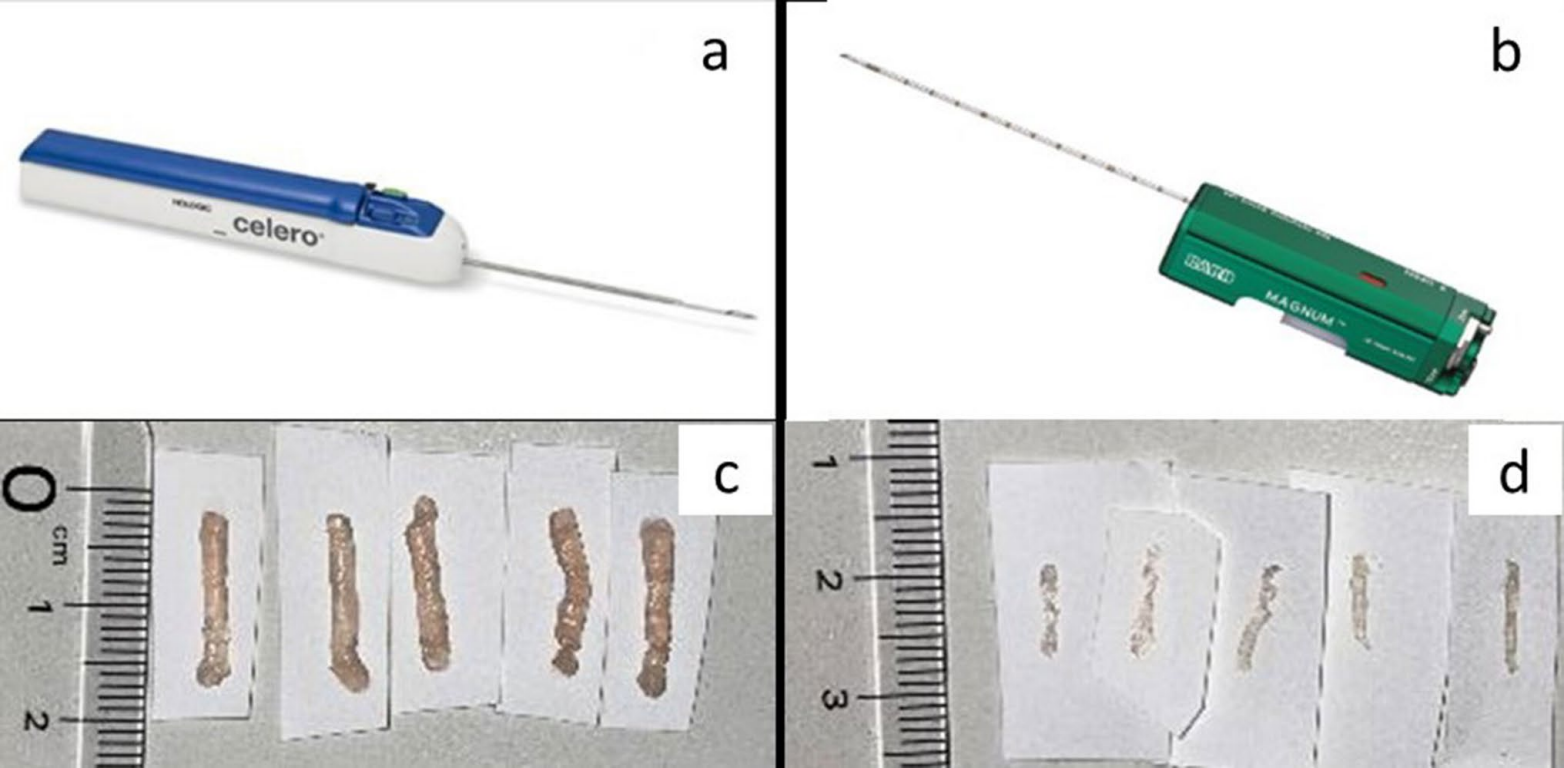
Core needle biopsy and vacuum-assisted biopsy devices and the specimens sampled from chicken phantoms with these devices. A larger volume of chicken-phantom specimens can be collected with the 12-gauge spring-loaded vacuum-assisted biopsy (VAB) device than with the 16-gauge spring-loaded core needle biopsy (CNB). **a** The 16-gauge spring-loaded CNB device (Magnum^™^). **b** The 12-gauge spring-loaded VAB device (Celero^®^). **c** Chicken-phantom specimens sampled with the 16-gauge spring-loaded CNB device (Magnum^™^). **d** Chicken-phantom specimens sampled with the 12-gauge spring-loaded VAB device (Celero^®^)

CNB was performed using a 16-gauge spring-loaded device (Magnum™; Bard, Inc., Tempe, AZ, USA) with a 22-mm throw following infiltration with 1% xylocaine. VAB was performed using a 12-gauge spring-loaded device (Celero^®^; Hologic Inc., Bedford, MA, USA) with 1% xylocaine and epinephrine. In our institution, the biopsies were usually performed in one of these two ways during the research period. Both CNB and VAB were typically performed two or three times; however, an additional sampling was performed if the sample volume was inadequate. Figure 1 shows the Magnum™ 16-gauge spring-loaded CNB device, the Celero^®^ 12-gauge spring-loaded VAB device, and the specimens of chicken phantoms sampled with these devices, respectively.

### Pathological evaluation

CNB and VAB specimens were immediately placed in 10% formalin and embedded in paraffin after fixation. The samples were cut into 3-μm-thick slices and then stained with hematoxylin–eosin. Immunohistochemical staining [hormone status: estrogen receptor, progesterone receptor, and human epidermal growth factor receptor (HER)-2] was performed at the discretion of the pathologists. Specimens were evaluated by more than two pathologists.

High-risk lesions included flat epithelial atypia, atypical ductal hyperplasia, lobular neoplasia (atypical lobular neoplasia and lobular carcinoma in situ), radial scars (radial sclerosing lesions, scleroelastotic lesions, sclerosing papillary lesions, and complex sclerosing lesions), papillary lesions (benign or atypical), and mucocele-like lesions [13].

### Statistical analysis

The chi-squared test for categorical variables and the Mann–Whitney *U* test for continuous variables were used to evaluate the associations of the biopsy device with imaging findings, clinical features, and histopathological results. The positive biopsy rate assessed as the positive predictive value 3 (PPV3) was calculated for each biopsy device. PPV3 is calculated by dividing the number of breast cancers by the number of biopsies (PPV3 = number of breast cancers/biopsies). PPV3 is widely used in Europe and the United States as an objective breast imaging quality measure. The rates of specimen failure, re-biopsy, upgrading from DCIS to invasive ductal carcinoma (IDC), and upgrading from high-risk to malignant lesions between the two devices were calculated and compared. In addition to the overall analysis including both masses and non-mass lesions, a subanalysis was performed including only non-mass lesions.

All calculations were performed using the Statistical Package for the Social Sciences (SPSS), version 24 (IBM SPSS Statistics for Windows, IBM Corp., Armonk, NY, USA). *P* values < 0.05 were accepted as indicating statistical significance.

## Results

Among the initial 465 patients with 512 lesions, 378 lesions underwent CNB and 134 lesions underwent VAB. However, five patients with five lesions who underwent CNB and seven patients with seven lesions who underwent VAB that were diagnosed as benign or high-risk lesions but were followed up at other hospitals were excluded, because their clinical course was unknown. Among the final 453 patients, 373 (74.6%) lesions that underwent CNB and 127 (25.4%) that underwent VAB were ultimately included in our study. Forty patients had two breast lesions, two patients had three breast lesions, and one patient had four breast lesions. Table 1 shows the histopathological results of the breast lesions in CNB and VAB.

**Table 1:** Histopathological results of breast lesions for core needle biopsy and vacuum-assisted biopsy

CNB (*n*)		VAB (*n*)	
Benign (117)	Malignant (231)	Benign (25)	Malignant (92)
Fibroadenoma (87)	Ductal carcinoma in situ (25)	Fibroadenoma (4)	Ductal carcinoma in situ (34)
Intraductal papilloma (13)	Invasive ductal carcinoma (166)	Intraductal papilloma (5)	Invasive ductal carcinoma (52)
Mastopathy (5)	Mucinous carcinoma (5)	Mastopathy (7)	Mucinous carcinoma (1)
Hemangioma (1)	Invasive lobular carcinoma (15)	Adenosis (1)	Invasive lobular carcinoma (3)
Mastitis (2)	Apocrine carcinoma (5)	Mastitis (1)	Apocrine carcinoma (2)
No malignancy (9)	Invasive micropapillary carcinoma (2)	No malignancy (7)	
	Malignant lymphoma (2)		
	Phyllodes tumor (malignant) (1)		

*CNB* core needle biopsy, *VAB* vacuum-assisted biopsy

Table 2 shows the clinical and pathological characteristics of breast lesions associated with CNB and VAB. There were 416 mass lesions and 84 non-mass lesions. The non-mass lesions were biopsied more frequently with VAB (57/127; 47.4%) than with CNB (27/378; 7.14%) (*P* = 0.000). There were no significant differences in patient age, lesion size, or number of samplings between CNB and VAB. Seven lesions were BI-RADS category 3, 363 lesions were category 4, and 130 lesions were category 5. All category 3 lesions were biopsied by CNB. CNB had a significantly higher percentage of category 5 lesions, and VAB had a significantly higher percentage of category 4 lesions (*P* = 0.000). The PPV3 was significantly higher for VAB (92/127; 72.4%) than for CNB (231/373; 61.9%) (*P* = 0.032).

**Table 2:** Clinical and pathological characteristics of breast lesions associated with core needle biopsy and vacuum-assisted biopsy

	All (*n* = 500)	CNB (*n* = 373)	VAB (*n* = 127)	*p*
Age (y)	56.2 ± 14.9	56.6 ± 15.5	55.2 ± 13.0	0.287
Mass (*n*)	416	346 (92.9%)	70 (52.6%)	0.000
Non-mass (*n*)	84	27 (7.1%)	57 (47.4%)	
Size (mm)	18.8 ± 15.4	18.4 ± 16.0	19.8 ± 13.5	0.241
BI-RADS Category 3	7	7 (1.9%)	0 (0%)	0.014
BI-RADS Category 4	363	259 (69.4%)	104 (81.9%)*	
BI-RADS Category 5	130	107 (28.7%)*	23 (18.1%)	
Number of samplings (*n*)	3.25 ± 0.70	3.24 ± 0.68	3.30 ± 0.76	0.308
Benign (*n*)	142	117 (31.4%)*	25 (19.7%)	0.000
Malignant (*n*)	323	231 (61.9%)	92 (72.4%)*	
[DCIS (*n*)]	[59]	[25 (6.7%)]	[34 (26.8%)]*	
High risk (*n*)	29	20 (5.4%)	9 (7.1%)	
Specimen failure (*n*)	6	5 (1.34%)	1 (0.78%)	
Positive biopsy rate (PPV3)	323 (64.6%)	231 (61.9%)	92 (72.4%)	0.032

Underlines are shown for *p*-values less than 0.05

Age, size, and number of samplings are shown as mean ± standard deviation

*CNB* core needle biopsy, *VAB* vacuum-assisted biopsy, *BI-RADS* breast imaging reporting and data system, *DCIS* ductal carcinoma in situ, *PPV3* positive predictive value 3

^*^significant difference

Among the specimen failures, 5/373 (1.34%) lesions (all the mass type) had undergone CNB, four had additional VAB, and one had additional excisional biopsy. Three of these lesions were ultimately diagnosed as malignant and two as benign. On the other hand, one lesion (0.78%) of 127 was a specimen failure with VAB; this lesion was a non-mass type, an excisional biopsy was added, and the lesion was diagnosed as malignant.

Table 3 shows the rates of re-biopsy and pathological upgrades for CNB and VAB. The lesion re-biopsy rate was 18/373 (4.82%) for CNB, 13 lesions had additional VAB, and five had additional excisional biopsy. The lesion re-biopsy rate was 3/127 (2.36%) for VAB, and all three lesions had an additional excisional biopsy.

**Table 3:** Rates of re-biopsy and pathological upgrade for core needle biopsy and vacuum-assisted biopsy

	All	CNB	VAB	*P*
Re-biopsy (*n*)	21/500	18/373 (4.82%)	3/127 (2.36%)	0.232
Upgrade from DCIS to IDC (*n*)	22/51	11/21 (52.4%)	11/30 (36.7%)	0.265
Upgrade from high risk to malignant (*n*)	6/27	5/19 (26.3%)	1/8 (12.5%)	0.043

*CNB* core needle biopsy, *VAB* vacuum-assisted biopsy, *DCIS* ductal carcinoma in situ, *IDC* invasive ductal carcinoma

Of the 59 lesions diagnosed as DCIS based on biopsy, 51 were surgically treated at our institution. The upgrade rate from DCIS to IDC was 11/21 (52.4%) for CNB and 11/30 (36.7%) for VAB. Of the 29 lesions diagnosed as high risk on biopsy, 27 were treated or followed up at our institution; of the 19 lesions that underwent CNB, six were followed up, eight had additional VAB, and four had additional excisional biopsy. Finally, 5/19 lesions (23.6%) were upgraded from high risk to malignant. On the other hand, of the eight lesions that underwent VAB, five were followed up and three underwent additional resection biopsy. Ultimately, 1/8 lesions (12.5%) were upgraded from high risk to malignant.

The high-risk to malignant lesion upgrade rate was significantly higher for CNB than for VAB. Although the rates of specimen failure, re-biopsy, and upgrade from DCIS to IDC tended to be higher for CNB, the differences were not significant.

Table 4 shows the clinical and pathological characteristics of non-mass lesions associated with CNB and VAB. There was no significant difference between the two in terms of BI-RADS category. The percentage of DCIS was significantly higher for VAB (25/57; 43.9%) than for CNB (5/27; 18.5%). The PPV3 was significantly higher for VAB (40/57; 70.2%) than for CNB (12/27; 44.4%) (*P* = 0.023). Although statistical analysis was not available due to the small sample size, the rates of re-biopsy and upgrade from high risk to malignant tended to be higher with CNB.

**Table 4:** Characteristics of non-mass lesions associated with core needle biopsy and vacuum-assisted biopsy

	All (*n* = 84)	CNB (*n* = 27)	VAB (*n* = 57)	*p*
BI-RADS Category 3	0	0 (0%)	0 (0%)	0.455
BI-RADS Category 4	78	26 (96.3%)	52 (91.2%)	
BI-RADS Category 5	6	1 (3.7%)	5 (8.8%)	
Benign (*n*)	23	10 (37.0%)	13 (19.7%)	0.045
Malignant (*n*)	52	12 (44.4%)	40 (70.2%)*	
[DCIS (*n*)]	[30]	[5 (18.5%)]	[25 (43.9%)]*	
High risk (*n*)	8	5 (18.5%)	3 (5.3%)	
Specimen failure (*n*)	1	0 (0%)	1 (1.8%)	
Positive biopsy rate (PPV3)	52 (61.9%)	12 (44.4%)	40 (70.2%)	0.023
Re-biopsy (*n*)	6/84	4/27 (14.8%)	2/57 (3.5%)	
Upgrade from DCIS to IDC (*n*)	11/25	2/4 (50.0%)	9/21 (42.9%)	
Upgrade from high risk to malignant (*n*)	2/8	2/5 (40.0%)	0/3 (0.0%)	

Underlines are shown for *p*-values less than 0.05

*CNB* core needle biopsy, *VAB* vacuum-assisted biopsy, *BI-RADS* breast imaging reporting and data system, *DCIS* ductal carcinoma in situ, *IDC* invasive ductal carcinoma, *PPV3* positive predictive value 3

^*^significant difference

**Fig. 2:**
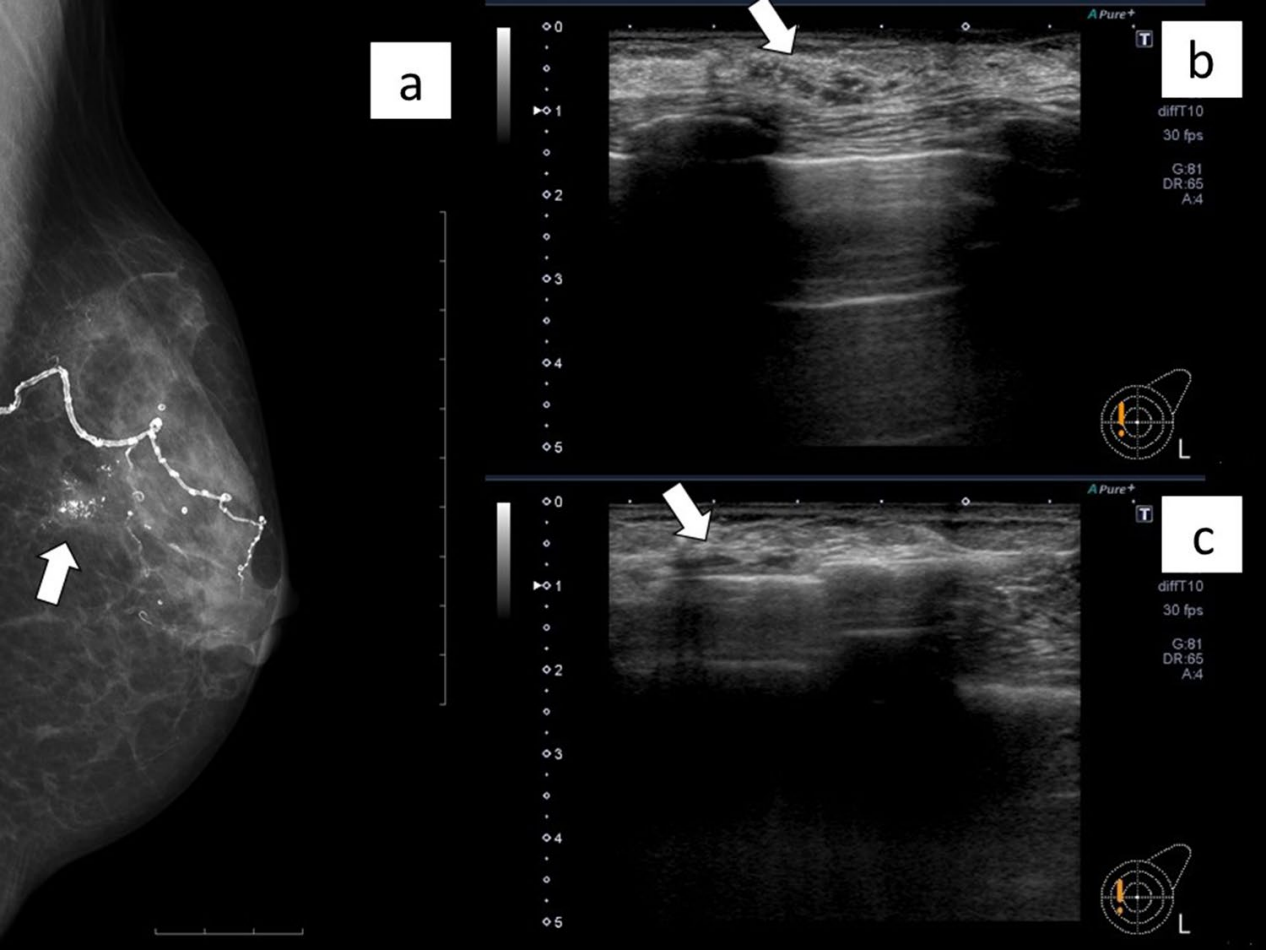
Non-mass lesion (ductal carcinoma in situ) diagnosed using vacuum-assisted biopsy. A woman in her 80s with calcified lesions on screening mammography. Mammogram showing clusters of pleomorphic calcifications in the left breast (arrow) (**a**). Ultrasonogram showing a 20-mm hypoechoic area with a punctate hypoechoic area corresponding to the calcifications, and the lesion was diagnosed as BI-RADS category 4 (arrow) (**b**). A 12-gauge spring-loaded vacuum-assisted biopsy was performed (arrow), and the lesion was diagnosed as ductal carcinoma in situ (**c**)

**Fig. 3:**
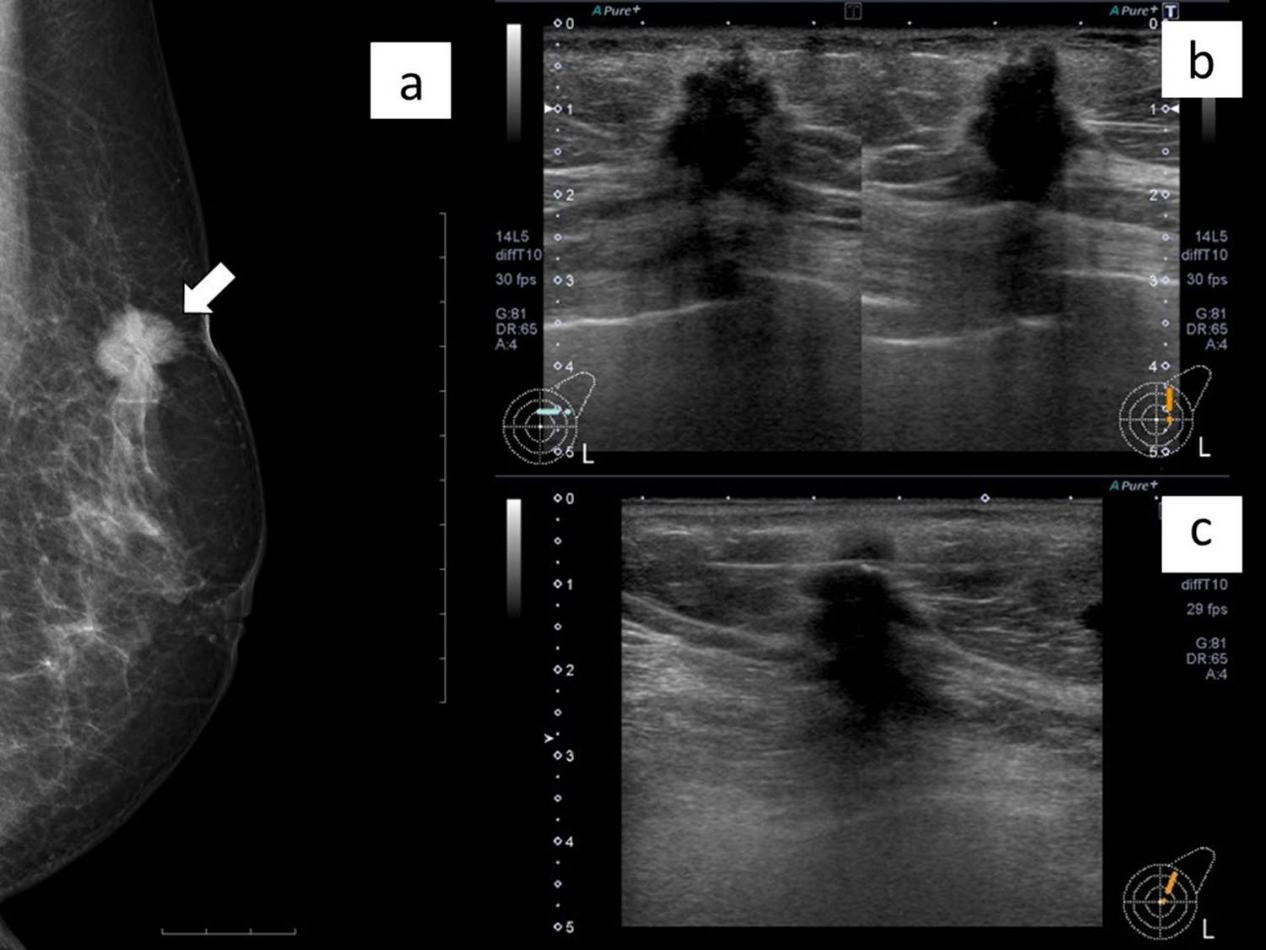
Mass (invasive ductal carcinoma) diagnosed using core needle biopsy. A woman in her 70s who was aware of a breast mass. Mammogram showing a spiculated mass with skin indentation in the right breast (arrow) (**a**). Ultrasonogram showing an irregularly shaped, indistinct mass 16 mm in diameter that was diagnosed as a BI-RADS category 5 lesion (**b**). A 16-gauge spring-loaded core needle biopsy was performed, and the lesion was diagnosed as invasive ductal carcinoma of the breast (**c**)

All patients who received CNB or VAB had no interruption of biopsy due to blood specks or pain. There were no cases of postoperative bleeding or pain requiring hospitalization. A representative case that underwent VAB is presented in Fig. 2, and a representative case that underwent CNB is shown in Fig. 3.

## Discussion

In this study, patients with breast lesions, including both masses and non-mass lesions who underwent US-guided spring-loaded 16-gauge CNB or 12-gauge spring-loaded VAB, were investigated. Although several papers have compared the usefulness of US-guided CNB and of US-guided VAB in the histological diagnosis of breast lesions, our study is the first to compare spring-loaded CNB with spring-loaded VAB.

Our study showed that US-guided VAB was performed more frequently than CNB for non-mass and BI-RADS 4 lesions (*P* = 0.000 and 0.000, respectively). Furthermore, the PPV3 was significantly higher for VAB (72.4%) than for CNB (61.9%) (*P* = 0.032). The choice between US-guided CNB and VAB was left to the discretion of the radiologist, resulting in a selection bias. This selection bias strongly influenced the results of the VAB and CNB comparisons, which necessitates careful consideration of these results.

Since the choice between CNB and VAB was left to the radiologists in this study, the selection bias of the operators was statistically evaluated. The first factor contributing to the operator's choice is that the accuracy for diagnosis of non-mass lesions is known to be lower for CNB than for VAB [6, 14]. Histologically, non-mass lesions are often heterogeneous and often confirmed as DCIS or invasive lobular carcinoma in malignant cases and mastopathy or sclerosing adenosis in benign cases [5, 6]. More specimens are needed to accurately diagnose these lesions. The second factor is that VAB tends to be selected in cases in which the US image does not clearly distinguish between benign and malignant (= BI-RADS 4) as VAB is more suitable for collecting a large amount of tissue, and CNB is selected in cases in which the lesion is most likely a malignant breast tumor (= BI-RADS 5). Previous studies have reported that VAB tends to be selected in BI-RADS 3 or 4 cases and CNB in BI-RADS 4 or 5 cases, which is consistent with our results [14].

We found that CNB diagnosed a higher percentage of fibroadenomas, which may indicate that CNB is more appropriate for diagnosis when the tumor is suggestive of being benign such as fibroadenoma and was performed appropriately by the operators in this study.

The less-invasive CNB is more appropriate for diagnosing fibroadenomas. On the other hand, VAB was found to diagnose a higher percentage of DCIS, which is usually depicted as a non-mass lesion, and VAB may be appropriate for such lesions. In fact, VAB was often selected for non-mass lesions, and a higher percentage of the lesions were pathologically diagnosed as DCIS. For this reason, the PPV3 may have been significantly higher for VAB than for CNB.

Previous reports showed that the PPV3 was 33.8% for patients examined for screening and 39.5% for patients examined for symptoms [15]. The PPV3 is an important indicator for assessing the quality of breast imaging examinations performed in breast specialty centers, and the PPV3 was higher in this study than in the previous studies. The reason is that our institution’s primary practice is to diagnose and treat breast cancer, and we do not perform much screening or follow-up of benign lesions. Most of our patients with symptoms and imaging findings that possibly indicate breast cancer are referred from nearby medical institutions.

The histological agreement between US-guided biopsy and surgical diagnosis is significantly lower in cases of non-mass lesions than of mass lesions [6]. The current study shows that even though non-mass lesions were indicated at a significantly higher percentage for VAB biopsies than for CNB, the upgrade rate from high-risk to malignant lesions was significantly higher for CNB than for VAB. Furthermore, there were no significant differences in the rates of specimen failure and re-biopsy between CNB and VAB, but upgrades from DCIS to IDC tended to be higher for CNB than for VAB. This study showed that the rate of upgrade from high-risk to malignant lesions was significantly higher for CNB (5/19; 26.3%) than for VAB (1/8; 12.5%). In a subanalysis including only non-mass lesions, the re-biopsy rate, upgrade rate from DCIS to IDC, and upgrade rate from high risk to malignant tended to be higher for CNB. These results suggest that when high-risk lesions are diagnosed using US-guided CNB, obtaining more specimens via US-guided VAB or surgical biopsy should be considered. On the other hand, even if a patient is diagnosed with a high-risk lesion based on US-guided VAB, follow-up may be possible if there is no discrepancy between the imaging findings and pathological results.

A larger volume of specimens can be obtained with VAB than with CNB, but VAB is more costly than CNB. Neither CNB nor VAB caused serious complications in this study. These results suggest that the 12-gauge spring-loaded VAB procedure is as safe as the 16-gauge spring-loaded CNB procedure. However, it is generally known that VAB is associated with a higher frequency of pain, hematoma, and other complications than CNB [9]. The optimal method should be selected after estimating the economic cost, patient burden, tissue type estimated from imaging findings, and the amount of specimen needed for diagnosis.

Some limitations of this study should be considered. First, the choice between US-guided CNB and VAB was left to the discretion of the radiologist, which caused selection bias. The selection bias strongly influenced the results of the comparative study between VAB and CNB. Second, this was a retrospective single-center study with a relatively small sample size. In the future, prospective large-scale studies will be required to examine the usefulness of US-guided sampling in patients with breast lesions. Third, two different US devices were used, and the procedures were performed by multiple radiologists. Because of the differences in image quality of the devices and skills of the radiologists, standardized images for analysis were not available, which may have affected the study results. However, since the two US systems were from the same vendor and the radiologists were well-trained specialists, the influence of the devices on the results should have been minimal.

## Conclusion

Although VAB had a significantly higher rate of biopsies of non-mass lesions, the rate of upgrade from high-risk to malignant lesions was significantly lower for VAB than for CNB. The rates of sample failure, re-biopsy, and upgrade from DCIS to IDC tended to be lower for VAB, although the differences were not significant. VAB may be more appropriate for biopsy of non-mass lesions.

## Data Availability

Not Applicable.
