# Correction: Comparison of the clinical and pathological characteristics of ultrasound-guided biopsy for breast masses and non-mass lesions between 16-gauge spring-loaded core needle biopsy and 12-gauge spring-loaded vacuum-assisted biopsy

**DOI:** 10.1007/s10396-025-01541-4

**Published:** 2025-04-15

**Authors:** Yuka Yashima, Tomoyuki Fujioka, Kazunori Kubota, Mio Mori, Arisa Sato, Goshi Oda, Tsuyoshi Nakagawa, Iichiroh Onishi, Mayuko Tanaka, Ukihide Tateishi

**Affiliations:** 1 Department of Diagnostic Radiology, Tokyo Medical and Dental University, 1-5-45 Yushima, Bunkyo-Ku, Tokyo, 113-8510 Japan; 2 Department of Radiology, Dokkyo Medical University Saitama Medical Center, 2-1-50 Minamikoshigaya, Koshigaya, Saitama 343-8555 Japan; 3 Department of Surgery, Breast Surgery, Tokyo Medical and Dental University, 1-5-45 Yushima, Bunkyo-Ku, Tokyo, 113-8510 Japan; 4 Department of Diagnostic Pathology, Tokyo Medical and Dental University, Tokyo, 113-8510 Japan; 5 Department of Radiology, Ome Municipal General Hospital, Tokyo, 198-0042 Japan

**Correction: Journal of Medical Ultrasonics (2023) 50:205–212** 10.1007/s10396-022-01279-3

The article “Comparison of the clinical and pathological characteristics of ultrasound‑guided biopsy for breast masses and non‑mass lesions between 16‑gauge spring‑loaded core needle biopsy and 12‑gauge spring‑loaded vacuum‑assisted biopsy”, written by Yuka Yashima, Tomoyuki Fujioka, Kazunori Kubota, Mio Mori, Arisa Sato, Goshi Oda, Tsuyoshi Nakagawa, Iichiroh Onishi, Mayuko Tanaka and Ukihide Tateishi, was originally published under exclusive license to The Author(s), under exclusive licence to The Japan Society of Ultrasonics in Medicine. As a result of the subsequent decision to publish the article under the open access model, the article’s copyright notice was changed on 25 March 2025 to © The Author(s) 2023 and the article is now distributed under a Creative Commons Attribution 4.0 International License, which permits use, sharing, adaptation, distribution and reproduction in any medium or format, as long as you give appropriate credit to the original author(s) and the source, provide a link to the Creative Commons licence, and indicate if changes were made. The images or other third party material in this article are included in the article’s Creative Commons licence, unless indicated otherwise in a credit line to the material. If material is not included in the article’s Creative Commons licence and your intended use is not permitted by statutory regulation or exceeds the permitted use, you will need to obtain permission directly from the copyright holder. To view a copy of this licence, visit http://creativecommons.org/licenses/by/4.0

The original article has been corrected.

